# Psychosocial Risk Factors in Cardiac Rehabilitation: Time to Screen Beyond Anxiety and Depression

**DOI:** 10.5334/gh.896

**Published:** 2021-02-19

**Authors:** Cristina Mesa-Vieira, Johannes Grolimund, Roland von Känel, Oscar H. Franco, Hugo Saner

**Affiliations:** 1 Graduate School for Health Sciences and Institute of Social and Preventive Medicine (ISPM), University of Bern, Bern, CH; 2 Psychosomatic Medicine Section, Department of Neurology, Bern University Hospital, CH; 3 Department of Consultation-Liaison Psychiatry and Psychosomatic Medicine, University Hospital Zurich, and University of Zurich, CH; 4 Department of Cardiology, Bern University Hospital, CH

**Keywords:** psychosocial risk factors, screening procedure, cardiovascular disease, cardiac rehabilitation, psychological counselling

## Abstract

**Background:**

Although it is well acknowledged that psychosocial risk factors (PSRF) such as low socio-economic status, stress, social isolation, negative emotions and negative personality patterns may contribute to the development and adverse outcome of cardiovascular disease (CVD), screening for PSRF in CVD patients is usually limited to anxiety and depression, mainly for feasibility reasons. We therefore aimed to develop a user-friendly screening battery for routine assessment of PSRFs and to evaluate this instrument regarding feasibility of application, PSRF results and attendance of psychological counselling if recommended to cardiac rehabilitation (CR) patients.

**Methods:**

This is a prospective single center cohort study including 609 consecutive CR patients. We first developed a screening instrument based on seven validated scales for the most relevant PSRFs with totally 90 questions presented in a uniform graphical design to facilitate completion called Psychocardiogram® (PCG) and applied the instrument in consecutive patients attending CR. Patients with positive screening results were invited to a psychological counseling session.

**Results:**

Six hundred and nine consecutive patients, aged 34 to 86 years (mean 60.7 years), 85% men, entering the CR program at the Bern University Hospital with ischemic heart failure (CHF), coronary artery disease (CAD) or peripheral artery disease, were included in this study. Eighty-three point three percent of the patients completed the PCG within 40 minutes. Vital exhaustion and Type-D personality were the most prevalent PSRFs (56.9% and 51.1%, respectively), whereas low social support (14.4%) and elevated depressive symptoms (15.9%), were the least prevalent ones. After screening, 120 patients (52.86%) with at least one PSRF made use of psychological counseling.

**Conclusions:**

We found the PCG to be a useful screening tool for PSRF in CR patients with the potential to get new insights into the prevalence of particular PSRF in specific populations and to better study their impact on occurrence and outcome of CVD.

## Introduction

Psychosocial Risk Factors (PSRFs) is a generally used term referring to both the presence of distress, and the absence of positive psychological resources. PSRFs affect the prognosis of ischemic heart disease (IHD) and significantly compromise patients’ health-related quality of life (HRQoL) [1,2,3,4]. Recent studies show that a range of PSRFs such as depression [5,6,7], anxiety [7,8], vital exhaustion [9], anger and hostility [10], work stress [11,12], type-D personality [13], social isolation [14,15], and low social-economic status increase the risk of recurrent cardiac events as well as cardiac and all-cause mortality in patients with IHD [16,17]. Conversely, positive psychological resources, such as high social support, have been described as protective factors for IHD [18]. PSRFs exert their adverse influence on cardiac outcomes by promoting an unhealthy lifestyle and by reducing chances of successful cardiac risk factor modification [1,19]. They also contribute to decreased adherence to medical treatment regimens and moderate the effects of cardiac rehabilitation [19,20,21]. In addition, a recent study found that loneliness is associated with the onset of CVD and CVD-related hospital admissions. This association was determined to be independent from other risk factors, which could suggest that it influences CVD through its own pathways that involve the immune system, the sympathetic drive and the hypothalamic–pituitary–adrenal axis. More specifically, it is suggested that there is an autonomic co-activation, increased pro-inflammatory cytokines, high reactivity and increased diurnal output [13]. Type-D personality has been described as a causal factor for withdrawal from CR and for the onset of symptoms of anxiety and depression [22]. It is possible that inflammation may also be one mechanism linking Type-2 personality with CVD risk.

Based on these and other findings, the European guidelines on CVD prevention in clinical practice recommend that PSRFs should be assessed and tailored clinical management should be considered in order to enhance HRQoL and IHD prognosis [23]. Assessment methods include clinical interviews, questionnaires and standardized structured interviews [24]. Guidelines released by European and American Cardiology Societies and Associations recommend the use of validated scales such as the Patient Health Questionnaire (PHQ), the Beck Depression Inventory (BDI), the Hospital Anxiety and Depression Scale (HADS) and the State-Trait Anxiety Inventory (SSAI) to assess the presence of depression and anxiety; ENRIIHD Social Support Instrument (ESSI) to assess social support; the State-Trait Anger Scale (STAS) and the Cook & Medley Hostility Scale (Ho) to measure anger and hostility; the Type D Scale 14; and HeartQoL to assess quality of life [24]. Other types of recommended instruments are structured interviews, such as the WHO Composite Clinical International Diagnostic Interview (CIDI) for the diagnosis of depression and anxiety [25].

Comprehensive screening for PSRFs in CR is not routine yet and includes few PSRFs at best, such as depression, anxiety or HRQoL. Although tools to screen for PSRFs are widely used, they are usually applied by specialized mental health professionals and their use is not common among other healthcare professionals treating patients with IHD. Moreover, the scoring and interpretation of these scales could be complex and may require a significant amount of time and resources that professionals working in crowded healthcare settings cannot afford. An important drawback for clinical application is a wide variation in display and response format across the various PSRF questionnaires, making their use in daily routine unattractive, not to say cumbersome. Today, the main focus of screening for PSRF is on anxiety and depression [37]. There are many other well-known PSRF, but aspects of interactions between these PSRF and their importance for the CR short- and long-term success and for morbidity and mortality are less well known.

The aim of this study is to develop a new battery of scales for routine screening and to assess a wide range of PSRFs and resources in patients with IHD entering a rehabilitation program. Besides efficient screening, results should help to get new insights into the prevalence of particular PSRFs in specific populations and to better study their impact on occurrence and outcome of CVD.

## Methods

### Patient population

This is a prospective single-center cohort study including 609 consecutive patients during a period of two years. All patients participated in a 6- to 12-week outpatient CR program at the Bern University Hospital, Switzerland as part of the Swiss CARE Study [26,27]. Before patients agreed to participate in the CR program, they received written information about the possible scientific use of the routinely collected data by administrative staff. All patients who agreed to participate provided written informed consent to the study protocol that was approved by the local ethical committee. Patients were included in the study if they had an angiographically confirmed coronary 1-, 2-, or 3-vessel disease (minimal stenotic diameter of 50%) and if they had participated in the CR program for at least six weeks. All patients had stable IHD when entering CR. Demographic and medical data were obtained from hospital charts. Cardiologists performed physical exams before starting the program as well as at discharge of patients. These examinations included cycle ergometry, weight measurement, blood pressure assessment, lipid determination and self-reported monitoring of smoking habits [28]. In addition, compliance with cardioprotective medications (antiplatelet, anticoagulation, statins, beta-blockers, ACE-inhibitors and AT2 antagonists) was checked.

### Cardiac Rehabilitation Program

Patients participated in a standard CR program three times a week for 6 to 12 weeks [23]. The program included a 70-hour exercise training with a main focus on aerobic endurance training and relaxation sessions (i.e. progressive muscle relaxation). Aerobic activities included calisthenics, strength training, water gymnastics, Nordic walking, hiking and cycling. Additionally, patients had 21 hours of group lectures, where they were educated about IHD-related issues. Addressed topics during these sessions were cardiovascular risk factors, management of anxiety and depression, healthy diet and cooking, and smoking cessation.

### The ‘Psychocardiogram’

The aim of the study was to screen for a full spectrum of PSRFs. Thus, The Psychocardiogram® was developed (Inselspital-Stiftung. Bern; Marke Nr. 561190, August 31, 2016). It is a specific set of self-reporting questionnaires based on validated scales and intends to screen for clinically relevant levels of depressive symptoms (7-item depression subscale of the Hospital Anxiety and Depression Sale – HADS) [28]; clinically relevant levels of anxiety symptoms (7-item anxiety subscale of the Hospital Anxiety and Depression Sale – HADS) [28]; anger/hostility (23-item cynicism subscale of the Minnesota Multiphasic Personality Inventory – MMPI2) [29]; type D personality (7-item negative affectivity subscale and 7-item social inhibition subscale – DS14) [30]; vital exhaustion (9-item Maastricht Vital Exhaustion Questionnaire) [31]; lack of social support (7-item ENRICHED Social Support Instrument – ESSI) [32]; and chronic work stress (6-item Effort subscale, 11-item reward subscale and 6-item overcommitment subscale of the Effort-Reward Imbalance Scale – ERI) [33]. All these have been identified as the most relevant PSRFs for poor prognosis in patients with IHD and are potentially modifiable within the setting of CR [1,34]. Further information on the particular instruments and their characteristics are displayed in Table 1. All instruments are fully standardized, are available in different languages, have already been extensively used in cardiology settings, show good psychometric properties, provide reliable and valid information on the according PSRF and are easy to administer. The entire set consisted of 90 single questions and required approximately 40 minutes to being answered (estimate based on the time needed by those patients who decided to fill the PCG out on site in the CR program). In order to facilitate the use and completion of these numerous questions, the PCG was formatted in a uniform way by using the same letters and spaces in the questionnaire for all questions and answers. This allowed patients to rapidly move from one answer to the following question (see questionnaire and graphical output in supplemental material).

**Table 1:** Psychocardiogram (Set of questionnaires).

Risk factor	Psychometric assessment	Time to administer (min)	Clinical cut-off used	Cronbach-α

Depression	Hospital Anxiety and Depression Scale (HADS); 7 items (depression)	5	Sum score >10	.839
Anxiety	Hospital Anxiety and Depression Scale (HADS); 7 items (anxiety)	5	Sum score >10	.826
Anger/Hostiiity	Minnesota Multiphasic Personality inventory (MMPI)-2; 23 items (cynicism subscaie)	5	Z-score > 1 SD	.898
Type D Pattern	DS 14; 14 items (7 items Negative Affectivity (NA); 7 items Social Inhibition (SI))	5	NA s 10 andSI > 10	.869 (NA), .880 (SI)
Vital Exhaustion	Maastricht Vital Exhaustion Questionnaire; 9 items	5	Sum Score s 11	.836
Lack of Social Support	ENRICHED Social Support Instrument (ESSI); 7 items	10	Sum Score < 18	.911
Chronic Work Stress	Effort-Reward Imbalance (ERI); 23 items (6 items Effort (E); 11 items Reward (R); 6 items Overcommitment (OC))	5	E/R*0.545454 > 1 Z-Score > 1 SD	.843 (E) .920 (R) .827 (OC)

### Screening Procedure

**Figure 1:**
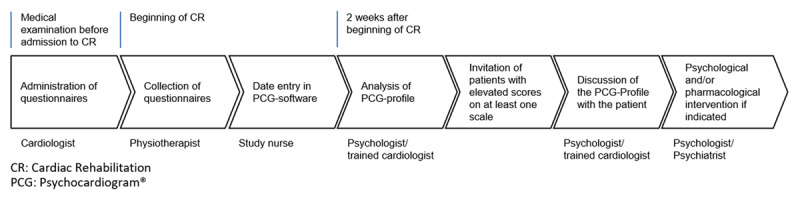
Screening procedure.

The screening process is shown in Figure 1. At the beginning of the CR program, all patients were routinely screened for PSRFs. At the time of the physical exam, each patient was given a specific set of questionnaires by the cardiologist and asked to fill them out onsite or if not feasible at home within the first week of the CR program. Completed questionnaires were then collected by the patient’s physical therapist. Data entry was performed by a study nurse. A designated Excel based program allowed for efficient entry and processing of all the relevant data, automatically computed scale values and z-scores and provided for each patient a graphical output displaying his or her individual PSRF profile, the Psychocardiogram® (see supplemental material). A psychologist/psychotherapist specialized in psychocardiology analyzed the results for each patient. If a patient met the criterion of at least one PSRF, based on a pre-specified cut-off score, he or she was invited by letter for a psychological consultation. The cardiologist informed the patient about the identified PSRF and its relevance for the prognosis of IHD, as well as for the patient’s quality of life. If indicated and patients agreed, this was followed by an in-depth assessment of psychological problems. Depending on the identified PSRF(s), as well as the result of the further diagnostic process, each patient was offered tailored psychological counseling, psychotherapy or psychopharmacological treatment.

### Data Analysis

Data was analyzed using PASW 17.0 statistical software package (SPSS Inc., Chicago, IL). Descriptive analysis was performed on demographic data (age, gender), number of patients effectively screened for PSRFs, number of patients positively screened for PSRFs, number of patients with identified PSRFs by screening attending a psychological counseling. T- and Spearmen X^2^- tests were performed on continuous and categorical variables respectively to assess demographic and psychosocial differences between patients who participated in at least a single psychological counseling session and those who did not. Cronbach’s Alpha was used to evaluate internal consistency of each of the PSRFs scales (Table 1). In addition, we evaluated whether the presence of PSRFs differed by gender and age.

## Results

### Patient characteristics

A total of 609 patients enrolled in the CR program at the Bern University Hospital between 2009 and 2011, met the inclusion criteria for the study. The mean age of the patients was 60 years (ranging between 34 and 86 years of age) and 517 (84.9%) of them were men. Of all the patients attending CR, 72 (12%) had a baseline diagnosis of ischemic heart failure; 505 (82.7%) had CAD (63% of them with a prior acute coronary event); and 32 (5.3%) patients presented a combination of CAD with significant peripheral arterial disease. There were no significant differences in the number of affected vessels among these patient groups. Table 2 gives an overview of the patients’ demographic and clinical characteristics at the beginning of the CR program.

**Table 2:** Demographic and clinical characteristics of patients included in the study (n = 609).

Variable	Patients (%)

Age* (years)	60.7 ± 10.9
Male gender,	84.9
Main diagnosis,	
Heart failure	12
Coronary artery disease (CAD)	82.7
CAD and peripheral artery disease (PAD)	5.3
CR program,	
CHF	11.9
DiaFit	2.4
Women	1.5
PAD	5.2
Seniors	13.1
Standard	65.9
Affected vessels,	
No affected vessels	2
One-vessel disease	34.8
Two-vessel disease	23.1
Three-vessel disease	33.1
N/A	7

### Screening process

**Figure 2:**
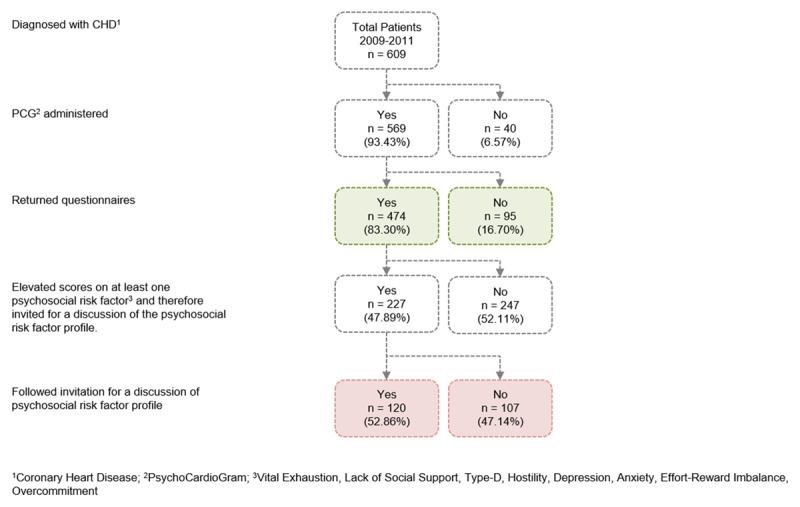
Screening process.

A schematic overview of the patient flow within the screening process is given in Figure 2. The screening instruments could be handed out to the majority of the patients (n = 569, 93.4%). Forty patients (6.6%) did not receive the questionnaires (due to refusal: n = 9, 22.5%; due to linguistic reasons: n = 25, 62.5%; and other not reported reasons: n = 6, 15%). Of those 569 patients, who received the questionnaires, 474 (83.3%) completed and returned them.

### Psychosocial risk factors

**Figure 3:**
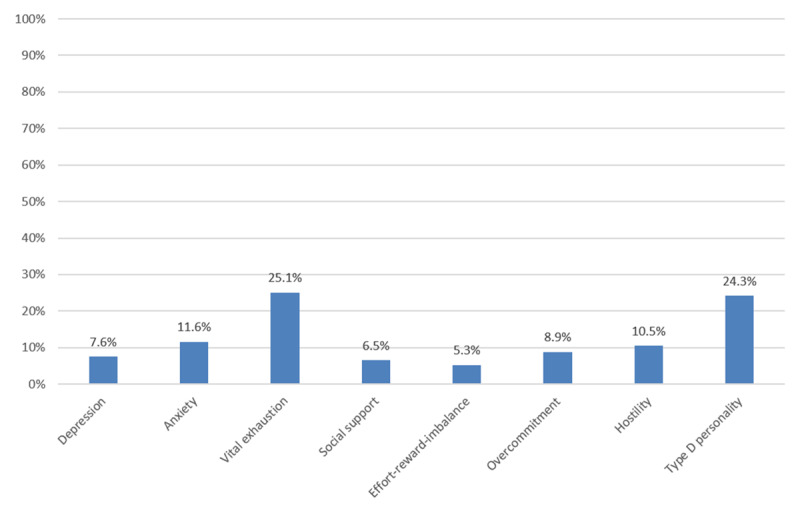
Percentage of patients with elevated scores per scale (n = 474).

Depression, anxiety and Type-D personality scales had the highest completion rates (n = 461 [97.2%] for depression and anxiety and n = 458 [96.6%] for type-D personality). ERI had the lowest completion rate (n = 197, 41.5%). Vital exhaustion and type-D personality were the most prevalent PSRFs among patients that returned the questionnaire (25.1% and 24.3%, respectively), whereas low social support (6.5%), elevated symptoms of depression (7.6%) were the least prevalent ones. Regarding overcommitment, 8.9% of the patients scored high, 11.6% had elevated symptoms of anxiety, 10.5% had increased hostility, and 5.3% reported low ERI. Only 107 patients completed the ERI scale because they were no longer employed and, therefore, had a very low prevalence in the group (Figure 3). Overall, 227 patients had one or more elevated PSRF score: whereas 39% presented with one PSRF, 27.3% had two, 16.7% three, 10.1% four, 5.3% five, 0.9% six risk factors and one patient (0.4%) had 7 risk factors. 227 patients (47.9%) screened positively for at least one PSRF and, therefore were invited for a psychological counseling.

### Psychological counselling attendance

After the initial screening, 120 patients (52.86%) made use of the psychological counseling. Table 3 shows the characteristics of patients stratified by their participation in psychological counseling with regard to identified PSRFs. All patients with an elevated score on the Psychocardiogram discussed their profile with the cardiologist. However, attendance to a counselling session was voluntary and almost half of the patients refused it. Reluctance to attend counselling was not further assessed, but regression analysis showed an association between being male, having higher hostility and effort-reward imbalance and not wanting to attend counselling.

**Table 3:** Sample characteristics of patients stratified by their participation in a psychological counseling with regard to identified psychosocial risk factors.

Variable	Following invitation discussion of the PCG-Profile		P Value*

	Yes (n = 120)	No (n = 107)	

Age, M ± SD, y	59.4 ± 10.1	60.3 ± 11.3	.569
Male gender, %	78.3	88.8	.035
Affected vessels, %			.076
One-vessel disease	35.3	40.2	
Two-vessel disease	28.4	15.5	
Three-vessel disease	36.2	44.3	
LVEF < 40%, %	20.8	16.8	.441
Acute coronary syndrome, %	63.3	62.3	.868
Depression score, M ± SD	6.2 ± 3.6	7.0 ± 4.3	.155
Anxiety score, M ± SD	7.6 ± 3.9	7.7 ± 4.0	.777
Vital exhaustion score, M ± SD	10.7 ± 4.8	10.2 ± 5.3	.504
Social support score, M ± SD	26.7 ± 6.8	26.2 ± 7.1	.582
Effort-reward-imbalance score, M ± SD	0.7 ± 0.3	0.9 ± 0.3	.053
Overcommitment score, M ± SD	15.4 ± 4.1	15.2 ± 4.5	.826
Hostility score, M ± SD	10.1 ± 6.4	12.0 ± 6.2	.028
Type D personality, %	50.0	52.4	.722

**M:** Mean. **SD:** Standard Deviation * P < 0.005.

## Discussion

We found the PCG to be a useful screening tool for PSRF in CR patients which allows new insights into the prevalence of particular PSRF in cardiac rehabilitation patients.

Targeting patients enrolled in a CR program over the course of two years provided us with a large sample of patients, spanning a wide age range between 34 and 86, with differing cumulative psychosocial experiences across a life span, assuming there was considerable heterogeneity regarding their psychosocial profile. The vast majority of the patients could be reached with the PCG, since the response rate of completed questionnaires was well over 80%. The use of the Psychocardiogram® among this cohort proves that it is possible to assess a wide range of PSRFs and positive resources in a relative short time (40 minutes on average). This shows that combining a set of established and widely available questionnaires in a well-structured and easy to depict graphical design, could be a valuable strategy to assess PSRFs and may also facilitate the development of individually tailored interventions.

In our study, vital exhaustion and type-D personality were the most prevalent PSRFs. Vital exhaustion (25.1%) and type-D personality (24.3%) were observed in about a quarter of all patients in our group. The lowest prevalence was found for low social support (6.5%) and elevated depressive symptoms (7.6%). Interestingly, depression has been one of the most studied PSRFs of cardiovascular disease and its role in the onset and prognosis of coronary heart disease is well established [6]. This finding could be explained by the relatively low prevalence of depression in the Swiss population, which is approximately 9% in persons aged 15 and older [35]. According to a recent systematic review and meta-analysis, vital exhaustion is associated with increased risk of fatal and non-fatal CAD and recurrent CAD events [9]. Some authors argue that somatic symptoms such as fatigue, anhedonia and irritability are found in persons with both vital exhaustion and depression and, therefore, psychometric scales might be overlapping [36]; although vital exhaustion is considered an independent factor for the onset of CAD. Meta-analysis may have explained the role of depression on the prediction of CHD on healthy populations, but the relationship between vital exhaustion and depression is yet to be ascertained. On the other hand, patients with type-D personality are more likely to develop depression after an acute coronary event due to their lack of planning and coping strategies [38].

Of patients approached with at least one PSRF, about half agreed on attending a session of psychological counselling. Further analysis showed that men and patients with increased hostility were less likely to attend psychological counselling. We did not explore personal reasons for the decision to decline the offer of psychological counselling in order to respect patients’ privacy.

Although there is no consensus on the tools that should be used to assess PSRFs among patients with CAD, there is considerable evidence for such a screening to be useful for patient care [34]. Other authors have also suggested the application of the Patient Health Questionnaire 9 (PHQ-9) and the General Anxiety Disorder scale 7 (GAD-7) for the evaluation of clinically relevant symptom levels of depression and anxiety respectively, instead of the HADS used in our study [1]. Further studies comparing the application of different scales in the context of cardiovascular disease and CR programs in particular are needed [39]. According to the 2016 European guidelines on cardiovascular disease prevention in clinical practice, besides the PSRF screened for in our study, it is also recommended to evaluate symptoms of post-traumatic stress disorder and other PSRFs that could interfere with treatment or the prognosis of the CAD [23].

Studies have been performed to assess the suitability of various screening questionnaires for depression in different populations. It could be argued that a reason for the low prevalence of depression found in our sample is attributed to the fact that HADS reflects the presence of general psychological distress rather than specific depressive symptoms [40], so the ability to detect a major depressive disorder depends on the severity of the symptoms. Sensitivity and specificity of HADS and BDI are similar, but the evidence has shown some variance across populations. In a study conducted by Strike et al. [41], the sensitivity of the HADS in patients one month after a myocardial infarction was 90%, while the sensitivity of BDI was 81.8%. However, Bunevicius et al. [42], found a sensitivity of 82% for HADS and 89% for BDI in patients with coronary artery disease undergoing cardiac rehabilitation. The European guidelines do not specify a tool for screening, but the first choice of the American Heart Association is the PHQ-9 and recommend BDI and HADS as adjunctive tools for screening [43]. In our study, HADS was the chosen tool for screening due to its ability to detect both anxiety and depressive symptoms at the same time and because it is shorter than the BDI.

Diagnosis of a psychiatric disorder can only be made by the means of a structured interview, which should be applied by a mental health professional or a trained person. However, not all cardiac rehabilitation settings have an interdisciplinary group that includes a mental health specialist. A recent survey about cardiac rehabilitation availability and delivery in Europe revealed large differences between regions and also compared with other high-income countries [46]. It is estimated that over 35% of the population in the United States live in an area with a shortage of mental health professional and, therefore, building a closer relationship between mental health providers and cardiologists is encouraged. Another advantage of the engagement of cardiologists in mental health is that it may help reduce stigma and improve the quality of life of the patients [43]. The use of electronic self-reported questionnaires can make screening for psychosocial risk factors easier and less time-consuming for all members of the cardiac rehabilitation team. Screening tools such as the Psychocardiogram have the potential to enable a fast and complete screening as opposed to the two-step approach [24].

Some studies have shown that depressive symptom severity can be reduced in patients undergoing CR [44]. Positive outcomes can also be achieved in terms of other PSRFs, including anxiety and anger [45]. However, the advantages of screening for PSRFs in CR are not restricted to the diagnosis of mental disorders and the facilitation of access to counselling and therapy. Screening can also be useful to optimize work processes in rehabilitation, to formulate patient-oriented goals, to measure therapy outcomes and to further investigate the relationship between CAD and PSRFs. Although there are some PSRFs that are more difficult to change if at all, including social isolation or some personality traits like hostility, screening results can guide health professionals at the CR setting in how to address these patients. Moreover, CR programs throughout the world are carried out differently; and involve diverse teams, in which psychologists or other mental health professionals are not always available. For instance, in Northern Europe, only 45.6% of the CR programs count with a psychologist as a team member [46].

Economic pressure on CR settings increases, as does the need of covering unmet goals in terms of access. It is estimated that CR demand will continue to increase due to demographic trends. CR programs are especially lacking in low- and middle-income countries, where the possibility of having large interdisciplinary CR groups are limited [47]. Even among member countries of the European Society of Cardiology there are significant differences in the duration of CR programs, team structures and formats. Therefore, including routine assessment strategies to screen for PSRFs that need to be performed by psychologists or psychiatrists could only be challenging for making efficient use of time, human and economic resources [48]. Another screening method is the so called two-step approach, which has been first proposed by Albus et al. [25], and which has been applied in the THORESCI study [49]. However, compared to our approach, this method seems time consuming and demands higher budgets. In contrast, many programs only use HADS and QoL questionnaires to assess depression, anxiety and quality of life to save time and resources. This approach, however, does not take into account the complexity of the PSRF profile and its effects in specific patients. Our results and the relative high rate of patients attending psychological counselling showed that our screening was efficient for the purpose of identifying potential PSRFs, although not able to diagnose psychiatric or psychological disorders. The question remains, whether having a structured interview would actually increase the turnout to psychological counselling. Similarly, it would also be interesting to see if categorizing patients by psychological profiles, as proposed by van Montfort and colleagues [49], would enhance treatment in the CR setting.

There may be benefits of psychosocial interventions in cardiac rehabilitation programs not only in regard to an improvement of the emotional status of the patients but also in the multidisciplinary team itself. Although we did not systematically evaluate this aspect, we have observed a much greater awareness and an increasing interest in psychosocial risk factors and their impact on patients’ involvement and participation by all team members.

Despite the evidence supporting the importance of screening for PSRFs, not many studies have reported their results with such a comprehensive set of scales in a large number of patients in a clinical setting, which is a strength of our study. Nevertheless, there is no consensus on the psychological interventions that will improve cardiac outcomes most effectively in patients undergoing CR [50]. Therefore, the prognostic consequences of screening for PSRFs need further investigation.

The main limitation of our study is that all scales have been independently validated in previous studies, but the battery as a whole has not be validated in any cardiac population. In addition, the study was conducted in one cardiac rehabilitation setting only. Therefore, results of this study are not comparable to other populations. Despite the high Cronbach’s Alpha scores of each scale, internal validity of the whole questionnaire still needs to be assessed. Consequently, the external validity of the questionnaire should be proven by its implementation in patients with other types of cardiovascular disease or other chronic diseases such as cancer or rheumatic disorders. The fact that not all patients filled all questionnaires might raise questions on the construct validity of the whole questionnaire. Qualitative assessment of the implementation of the PCG in CR could help to understand acceptability among staff and patients.

## Conclusions

The PCG turned out to be a useful screening tool for PSRF in CR patients with the potential to get new insights into the prevalence of particular PSRF beyond anxiety and depression in specific populations and to better study their impact on occurrence and outcome of CVD. The PCG can be applied with minimal CR staff resources, has a high return rate and allows targeted recommendations for further professional evaluation. Although screening for PSRF can be easy and convenient by using the PCG, type and value of therapeutic interventions as well as their impact on prognosis has to be further evaluated.

## References

[1] Pedersen SS, von Kanel R, Tully PJ, et al. Psychosocial perspectives in cardiovascular disease. Eur J Prev Cardiol. 2017; 24: 108–115. 2017/06/18. DOI: 10.1177/2047487317703827 28618908

[2] Richards SH, Anderson L, Jenkinson CE, et al. Psychological interventions for coronary heart disease: Cochrane systematic review and meta-analysis. Eur J Prev Cardiol. 2018; 25: 247–259. 2017/12/08. DOI: 10.1177/2047487317739978 29212370

[3] Tusek-Bunc K, Petek D. Comorbidities and characteristics of coronary heart disease patients: Their impact on health-related quality of life. Health Qual Life Outcomes. 2016; 14: 159 2016/11/17. DOI: 10.1186/s12955-016-0560-1 27846850 PMC5111348

[4] Sajobi TT, Wang M, Awosoga O, et al. Trajectories of Health-Related Quality of Life in Coronary Artery Disease. Circ Cardiovasc Qual Outcomes. 2018; 11: e003661 2018/03/17. DOI: 10.1161/CIRCOUTCOMES.117.003661 29545392

[5] Dickens C. Depression in people with coronary heart disease: Prognostic significance and mechanisms. Curr Cardiol Rep. 2015; 17: 83 2015/08/19. DOI: 10.1007/s11886-015-0640-6 26277367

[6] Carney RM, Freedland KE. Depression and coronary heart disease. Nat Rev Cardiol. 2017; 14: 145–155. 2016/11/18. DOI: 10.1038/nrcardio.2016.181 27853162

[7] Shen BJ, Fan Y, Lim KSC, et al. Depression, Anxiety, Perceived Stress, and Their Changes Predict Greater Decline in Physical Health Functioning over 12 Months Among Patients with Coronary Heart Disease. Int J Behav Med. 2019; 26: 352–364. 2019/06/21. DOI: 10.1007/s12529-019-09794-3 31218559

[8] Lu Y, Jiang Y, Gu L. Using path analysis to investigate the relationships between depression, anxiety, and health-related quality of life among patients with coronary artery disease. Qual Life Res. 2019 2019/05/18. DOI: 10.1007/s11136-019-02207-8 31098799

[9] Frestad D, Prescott E. Vital Exhaustion and Coronary Heart Disease Risk: A Systematic Review and Meta-Analysis. Psychosom Med. 2017; 79: 260–272. 2016/12/03. DOI: 10.1097/PSY.0000000000000423 27902666

[10] Smaardijk VR, Lodder P, Kop WJ, et al. Sex- and Gender-Stratified Risks of Psychological Factors for Incident Ischemic Heart Disease: Systematic Review and Meta-Analysis. J Am Heart Assoc. 2019; 8: e010859 2019/04/30. DOI: 10.1161/JAHA.118.010859 31030598 PMC6512085

[11] Sara JD, Prasad M, Eleid MF, et al. Association Between Work-Related Stress and Coronary Heart Disease: A Review of Prospective Studies Through the Job Strain, Effort-Reward Balance, and Organizational Justice Models. J Am Heart Assoc. 2018; 7 2018/04/29. DOI: 10.1161/JAHA.117.008073 PMC6015274 29703810

[12] Wirtz PH, von Kanel R. Psychological Stress, Inflammation, and Coronary Heart Disease. Curr Cardiol Rep. 2017; 19: 111 2017/09/22. DOI: 10.1007/s11886-017-0919-x 28932967

[13] Kupper N, Denollet J. Type DPersonality as a Risk Factor in Coronary Heart Disease: A Review of Current Evidence. Curr Cardiol Rep. 2018; 20: 104 2018/09/14. DOI: 10.1007/s11886-018-1048-x 30209683 PMC6153564

[14] Hakulinen C, Pulkki-Raback L, Virtanen M, et al. Social isolation and loneliness as risk factors for myocardial infarction, stroke and mortality: UK Biobank cohort study of 479 054 men and women. Heart. 2018; 104: 1536–1542. 2018/03/29. DOI: 10.1136/heartjnl-2017-312663 29588329

[15] Valtorta NK, Kanaan M, Gilbody S, et al. Loneliness and social isolation as risk factors for coronary heart disease and stroke: Systematic review and meta-analysis of longitudinal observational studies. Heart. 2016; 102: 1009–1016. 2016/04/20. DOI: 10.1136/heartjnl-2015-308790 27091846 PMC4941172

[16] Bhatnagar A. Environmental Determinants of Cardiovascular Disease. Circ Res. 2017; 121: 162–180. 2017/07/08. DOI: 10.1161/CIRCRESAHA.117.311811 28684622 PMC5777598

[17] Tang KL, Rashid R, Godley J, et al. Association between subjective social status and cardiovascular disease and cardiovascular risk factors: A systematic review and meta-analysis. BMJ Open. 2016; 6: e010137 2016/03/20. DOI: 10.1136/bmjopen-2015-010137 PMC4800117 26993622

[18] Barth J, Schneider S, von Kanel R. Lack of social support in the etiology and the prognosis of coronary heart disease: A systematic review and meta-analysis. Psychosom Med. 2010; 72: 229–238. 2010/03/13. DOI: 10.1097/PSY.0b013e3181d01611 20223926

[19] Pogosova N, Kotseva K, De Bacquer D, et al. Psychosocial risk factors in relation to other cardiovascular risk factors in coronary heart disease: Results from the EUROASPIRE IV survey. A registry from the European Society of Cardiology. Eur J Prev Cardiol. 2017; 24: 1371–1380. 2017/05/24. DOI: 10.1177/2047487317711334 28534422

[20] Crawshaw J, Auyeung V, Norton S, et al. Identifying psychosocial predictors of medication non-adherence following acute coronary syndrome: A systematic review and meta-analysis. J Psychosom Res. 2016; 90: 10–32. 2016/10/25. DOI: 10.1016/j.jpsychores.2016.09.003 27772555

[21] Kessing D, Denollet J, Widdershoven J, et al. Psychological Determinants of Heart Failure Self-Care: Systematic Review and Meta-Analysis. Psychosom Med. 2016; 78: 412–431. 2016/04/16. DOI: 10.1097/PSY.0000000000000270 27082055

[22] Kupper N, Pedersen SS, Hofer S, et al. Cross-cultural analysis of type D (distressed) personality in 6222 patients with ischemic heart disease: A study from the International HeartQoL Project. Int J Cardiol. 2013; 166: 327–333. 2011/11/15. DOI: 10.1016/j.ijcard.2011.10.084 22078395

[23] Piepoli MF, Hoes AW, Agewall S, et al. 2016 European Guidelines on cardiovascular disease prevention in clinical practice: The Sixth Joint Task Force of the European Society of Cardiology and Other Societies on Cardiovascular Disease Prevention in Clinical Practice (constituted by representatives of 10 societies and by invited experts) Developed with the special contribution of the European Association for Cardiovascular Prevention & Rehabilitation (EACPR). Eur Heart J. 2016; 37: 2315–2381. 2016/05/26. DOI: 10.1007/s12529-016-9583-6 27222591 PMC4986030

[24] Albus C, Jordan J, Herrmann-Lingen C. Screening for psychosocial risk factors in patients with coronary heart disease: Recommendations for clinical practice. European Journal of Cardiovascular Prevention & Rehabilitation. 2004; 11: 75–79. DOI: 10.1097/01.hjr.0000116823.84388.6c 15167210

[25] Jackson AC, Le Grande MR, Higgins RO, et al. Psychosocial Screening and Assessment Practice within Cardiac Rehabilitation: A Survey of Cardiac Rehabilitation Coordinators in Australia. Heart Lung Circ. 2017; 26: 64–72. 2016/06/11. DOI: 10.1016/j.hlc.2016.04.018 27283446

[26] Stauber S, Schmid JP, Saner H, et al. A comparison of psychosocial risk factors between 3 groups of cardiovascular disease patients referred for outpatient cardiac rehabilitation. J Cardiopulm Rehabil Prev. 2012; 32: 175–181. 2012/03/20. DOI: 10.1097/HCR.0b013e31824cc1f7 22426505

[27] Blum MR, Schmid JP, Eser P, et al. Long-term results of a 12-week comprehensive ambulatory cardiac rehabilitation program. J Cardiopulm Rehabil Prev. 2013; 33: 84–90. 2013/02/07. DOI: 10.1097/HCR.0b013e3182779b88 23385556

[28] Hermann C, Buss U. Vorstellung und Validierung einer deutschen Version der Hospital Anxiety and Depression Scale (HAD-Skala): Ein Fragebogen zur Erfassung des psychischen Befindens bei Patienten mit körperlichen Beschwerden. Diagnostica. 1994; 40: 143–154.

[29] Moultrie JK, Engel RR. Empirical Correlates for the Minnesota Multiphasic Personality Inventory-2-Restructured Form in a German Inpatient Sample. Psychol Assessment. 2017; 29: 1273–1289. DOI: 10.1037/pas0000415 27918175

[30] Denollet J. DS14: Standard assessment of negative affectivity, social inhibition, and Type D personality. Psychosom Med. 2005; 67: 89–97. 2005/01/28. DOI: 10.1097/01.psy.0000149256.81953.49 15673629

[31] Kudielka BM, von Kanel R, Preckel D, et al. Exhaustion is associated with reduced habituation of free cortisol responses to repeated acute psychosocial stress. Biol Psychol. 2006; 72: 147–153. 2005/10/21. DOI: 10.1016/j.biopsycho.2005.09.001 16236419

[32] Kendel F, Spaderna H, Sieverding M, et al. Eine deutsche Adaptation des ENRICHD Social Support Inventory (ESSI). Diagnostica. 2011; 57: 99–106. DOI: 10.1026/0012-1924/a000030

[33] Siegrist J, Wege N, Puhlhofer F, et al. A short generic measure of work stress in the era of globalization: Effort-reward imbalance. Int Arch Occup Environ Health. 2009; 82: 1005–1013. 2008/11/20. DOI: 10.1007/s00420-008-0384-3 19018554

[34] Pogosova N, Saner H, Pedersen SS, et al. Psychosocial aspects in cardiac rehabilitation: From theory to practice. A position paper from the Cardiac Rehabilitation Section of the European Association of Cardiovascular Prevention and Rehabilitation of the European Society of Cardiology. Eur J Prev Cardiol. 2015; 22: 1290–1306. 2014/07/26. DOI: 10.1177/2047487314543075 25059929

[35] BFS. Psychische Gesundheit. 2019 (accessed 02/07/2020).

[36] Kop WJ. Somatic depressive symptoms, vital exhaustion, and fatigue: Divergent validity of overlapping constructs. Psychosom Med. 2012; 74: 442–445. 2012/06/12. DOI: 10.1097/PSY.0b013e31825f30c7 22685237

[37] Van der Kooy K, van Hout H, Marwijk H, et al. Depression and the risk for cardiovascular diseases: Systematic review and meta analysis. Int J Geriatr Psychiatry. 2007; 22: 613–626. 2007/01/20. DOI: 10.1002/gps.1723 17236251

[38] Yamaguchi D, Izawa A, Matsunaga Y. The Association of Depression with Type D Personality and Coping Strategies in Patients with Coronary Artery Disease. Intern Med. 2020; 59: 1589–1595. 2020/07/03. DOI: 10.2169/internalmedicine.3803-19 32612062 PMC7402968

[39] Thombs BD, Roseman M, Coyne JC, et al. Does evidence support the American Heart Association’s recommendation to screen patients for depression in cardiovascular care? An updated systematic review. PLoS One. 2013; 8: e52654 2013/01/12. DOI: 10.1371/journal.pone.0052654 23308116 PMC3538724

[40] Westhoff-Bleck M, Winter L, Aguirre Davila L, et al. Diagnostic evaluation of the hospital depression scale (HADS) and the Beck depression inventory II (BDI-II) in adults with congenital heart disease using a structured clinical interview: Impact of depression severity. Eur J Prev Cardiol. 2020; 27: 381–390. 2019/07/28. DOI: 10.1177/2047487319865055 31349778

[41] Strik JJ, Honig A, Lousberg R, et al. Sensitivity and specificity of observer and self-report questionnaires in major and minor depression following myocardial infarction. Psychosomatics. 2001; 42: 423–428. 2001/12/12. DOI: 10.1176/appi.psy.42.5.423 11739910

[42] Bunevicius A, Staniute M, Brozaitiene J, et al. Diagnostic accuracy of self-rating scales for screening of depression in coronary artery disease patients. J Psychosom Res. 2012; 72: 22–25. 2011/12/28. DOI: 10.1016/j.jpsychores.2011.10.006 22200518

[43] Jha MK, Qamar A, Vaduganathan M, et al. Screening and Management of Depression in Patients With Cardiovascular Disease: JACC State-of-the-Art Review. J Am Coll Cardiol. 2019; 73: 1827–1845. 2019/04/13. DOI: 10.1016/j.jacc.2019.01.041 30975301 PMC7871437

[44] Rutledge T, Redwine LS, Linke SE, et al. A meta-analysis of mental health treatments and cardiac rehabilitation for improving clinical outcomes and depression among patients with coronary heart disease. Psychosom Med. 2013; 75: 335–349. 2013/05/01. DOI: 10.1097/PSY.0b013e318291d798 23630306

[45] Hazelton G, Williams JW, Wakefield J, et al. Psychosocial benefits of cardiac rehabilitation among women compared with men. J Cardiopulm Rehabil Prev. 2014; 34: 21–28. 2013/12/12. DOI: 10.1097/HCR.0000000000000034 24326900

[46] Abreu A, Pesah E, Supervia M, et al. Cardiac rehabilitation availability and delivery in Europe: How does it differ by region and compare with other high-income countries?: Endorsed by the European Association of Preventive Cardiology. Eur J Prev Cardiol. 2019; 26: 1131–1146. 2019/02/21. DOI: 10.1177/2047487319827453 30782007

[47] Gimigliano F, Negrini S. The World Health Organization ‘Rehabilitation 2030: A call for action.’ Eur J Phys Rehabil Med. 2017; 53: 155–168. 2017/04/07. DOI: 10.23736/S1973-9087.17.04746-3 28382807

[48] Ruivo JAA, Dendale P, et al. Overview of Cardiac Rehabilitation (OCRE) in ESC member countries. 2019 https://www.escardio.org/static_file/Escardio/Subspecialty/EAPC/Country%20of%20the%20month/Documents/OCRE%203.0_presentation_kit2019_Final.pdf (accessed 29/10/2019).

[49] van Montfort E, Denollet J, Vermunt JK, et al. The tense, the hostile and the distressed: Multidimensional psychosocial risk profiles based on the ESC interview in coronary artery disease patients – the THORESCI study. Gen Hosp Psychiatry. 2017; 47: 103–111. 2017/08/16. DOI: 10.1016/j.genhosppsych.2017.05.006 28807133

[50] Albus C, Herrmann-Lingen C, Jensen K, et al. Additional effects of psychological interventions on subjective and objective outcomes compared with exercise-based cardiac rehabilitation alone in patients with cardiovascular disease: A systematic review and meta-analysis. Eur J Prev Cardiol. 2019; 26: 1035–1049. 2019/03/13. DOI: 10.1177/2047487319832393 30857429 PMC6604240

